# ElasticPay: Instant Peer-to-Peer Offline Extended Digital Payment System

**DOI:** 10.3390/s24248034

**Published:** 2024-12-16

**Authors:** Annapureddy Venkata Sai Kumar Reddy, Gourinath Banda

**Affiliations:** Department of Computer Science and Engineering, Indian Institute of Technology Indore, Indore 453552, India

**Keywords:** central bank digital currency, offline mobile transaction, security, trusted platform module, attestation, privacy, double spending

## Abstract

The widespread reliance on paper-based currency poses significant drawbacks, such as counterfeiting, lack of transparency, and environmental impacts. While Central Bank Digital Currencies (CBDCs) address many of these issues, their dependence on continuous internet connectivity limits their usability in scenarios with poor or no network access. To overcome such limitations, this paper introduces ElasticPay, a novel Peer-to-Peer (P2P) Offline Digital Payment System that leverages advanced hardware security measures realised through Trusted Platform Modules (TPMs), Trusted Execution Environments (TEEs), and Secure Elements (SEs). ElasticPay ensures transaction privacy, unforgeability, and immediate settlement while preventing double spending. Our approach integrates robust recovery mechanisms and provides a scalable solution for diverse environments. Extensive experimentation validates the system’s reliability and practicality, highlighting its potential to advance secure and inclusive CBDC ecosystems. We demonstrate the proposed solution implementation on the iPhone mobilephone because it has an inbuilt Secure Enclave, which is an integrated implementation of the necessary TPM, TEE, and SE functionalities.

## 1. Introduction

The currency we know in modern society and businesses has transitioned over various stages starting from the barter system [1] for exchanging goods/services to commodity money such as gold and silver coins to paper-printed currency. However, paper currency has several drawbacks such as lack of transparency, excessive anonymity, and environmental impact, including carbon footprint [2] and counterfeiting issues [3]. In the last two decades, blockchain-based decentralized cryptocurrencies have appeared, fostering progress in areas such as governance, voting systems, supply chain management, decentralized autonomous organizations, and the creation of Central Bank Digital Currencies (CBDCs). A CBDC [4] is a digital currency issued by a centralised authority. CBDC is defined as a digital form of fiat money issued by a central bank, adhering to standard rules and overcoming the limitations of paper currency. CBDC offers several benefits [5,6,7] such as reduced operational costs, enhanced security, and increased transparency, contributing to financial inclusion and sustainability [3]. The paper-currency system offers digital services, also known as digital fiat currency (DFC), allowing balance transfers via telecom connectivity [3]. However, if connectivity is lost, reliance on paper currency ensures transaction success. With the advent of Central Bank Digital Currency (CBDC), a new challenge arises in situations where internet connectivity is either absent or poor; because of its fully digital and centralised nature, CBDC relies on active telecom/internet connectivity [3] for a transaction/transfer to occur. Internet or telecom connectivity remains a challenge not only in the developing/underdeveloped world but also, for various reasons, in the developed world. For example, frequent bank-server downtimes for maintenance, coupled with internet outages during natural disasters, exacerbate this issue [8]. Thus, such dependency on network connectivity limits spending freedom and hence prompts for an offline-payment transaction system to ensure uninterrupted transactions even without network connectivity [3].

Currently, there are two main methods for offline payments [9]: those that rely on smart cards [10] and those that use smartphones [11,12,13,14]. We have chosen to focus on smartphone-based payments for their clear advantages over the smart card approach, such as greater convenience and ease of carrying. In contrast, smart card transactions require both parties to have their cards and to use a Point of Sale (POS) terminal. This means that for any transaction to happen, a bulky terminal device is necessary, making it less practical since it is an additional item to carry around. Smartphone-based payments streamline this process, making transactions simpler and more accessible.

In response, we have developed a smartphone-based offline payment system called Peer-to-Peer Extended Digital Offline Payment System (‘ElasticPay’), which prioritizes security measures [9] and introduces an additional feature of *preserving user privacy in offline situations.* It is meticulously crafted to support transactions using both Central Bank Digital Currencies (CBDCs) and various forms of digital money. This dual compatibility ensures that our solution is versatile, future-proof, and backwards-compatible, thus catering to all types of digital transactions while ensuring security and privacy in offline environments. Privacy is the most sought-after feature in a digital euro [15], highlighting that the need for privacy extends beyond just this currency to all digital transactions. The main features of our system include comprehensive security measures [9], immediate settlement, extended support, privacy protection, and transaction failure recovery.

Throughout the remainder of this paper, the terms “telecom connectivity” and “internet connectivity” will be collectively referred to as “network connectivity” for brevity and clarity. This unified term encompasses all forms of telecommunications and internet connections that facilitate digital communication and transactions.

The main contributions of this paper are:

1. **Enhanced Privacy Protection**: We enhance the privacy of both the sender and receiver in offline payment transactions.
2. **Robust Security Features**: While Trusted Execution Environments (TEEs) are widely used to enhance security, we propose integrating the Trusted Platform Module (TPM) with TEE. This integration will further strengthen the system’s resilience, especially in offline environments, against various attacks.
3. **Comprehensive Explanation**: This paper provides a clear and detailed explanation of the mechanisms and processes involved in offline digital payment transactions, offering valuable insights into their implementation and benefits.

The rest of this paper is organized as follows: Section 2 gives preliminary concepts and definitions necessary for understanding the subsequent presentation about our proposed solution. The proposed offline payment system is presented in Section 3. Section 4 presents the experimental setup and particulars on experiments carried out. The related work is given in Section 5, while Section 6 concludes the paper with a discussion about the contributions of our work and directions for future research in enhancing digital payment systems.

## 2. Preliminaries

This section elaborates on the basic concepts and definitions that help readers understand subsequent discussions on our proposed solution called ElasticPay. The financial system is supported by three key stakeholders, and they are central banks, intermediary banks, and customers. The central bank issues currency, facilitates credit, regulates monetary policy, maintains financial stability, and oversees the banking system. Intermediary banks promote financial inclusivity, manage deposits, distribute loans, ensure regulatory compliance, provide payment services, and facilitate fund transfers. Ultimately, customers—individuals, businesses, and organizations—drive economic activity by conducting transactions via intermediary banks, including deposits, withdrawals, loans, payments, and investments. Throughout this paper, the term ‘bank’ is used to refer to an intermediary bank, while the term ‘central bank’ is used to refer specifically to the central bank.

**Figure 1:**
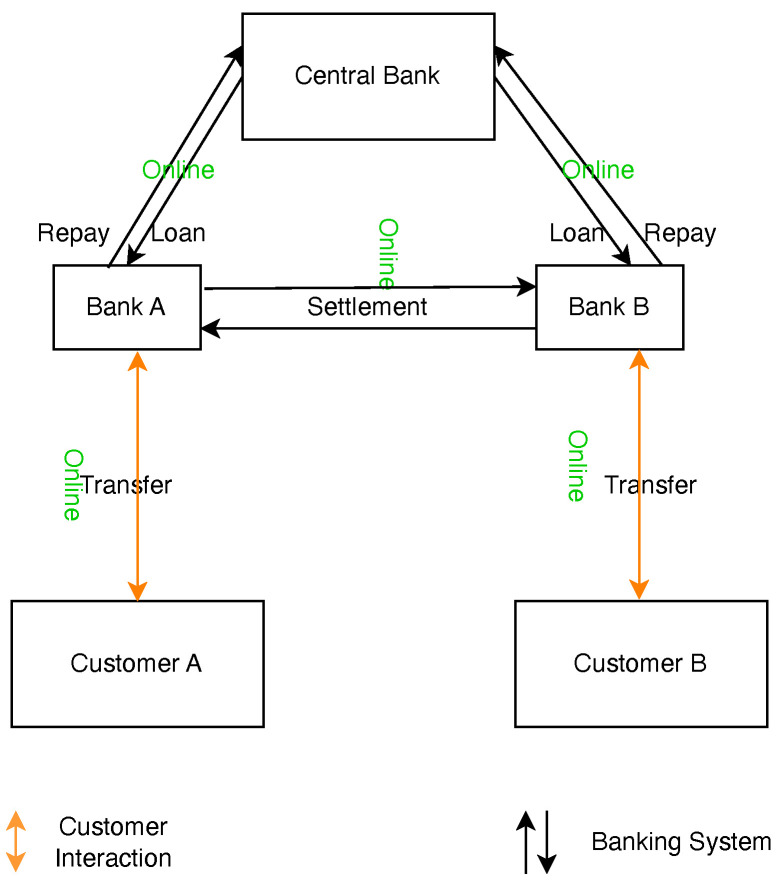
Online banking transaction.

In a typical online banking scenario, such as when a client (Customer A) at one bank wishes to send funds to another (Customer B) at a different bank, as depicted in Figure 1, the process follows these steps:

1. Initiation: The sender (Customer A) initiates the transaction through their bank’s digital platform.
2. Verification: The originating bank verifies transaction details against account status and customer permissions.
3. Interbank Communication: The transaction details are sent to a central processing system or directly to the recipient’s bank, which may involve interaction with the central bank if CBDCs are utilized.
4. Completion: Upon validation, the recipient’s (Customer B) bank credits their account, thereby concluding the transaction process.

Initiating an offline transaction requires a sufficient balance in the sender’s device-stored offline wallet, which is pre-funded by transferring funds from their primary bank account. To enable the exchange between sender and receiver, the system utilizes inherent wireless communication protocols like Bluetooth or NFC, obviating the need for a network connection, thus characterizing the truly offline nature of the payment process.

Online transactions typically involve intermediaries, but our focus is on offline payments, where only the sender and receiver are involved. This setup presents unique challenges, as it increases the potential for attacks. There is a chance that either party might intentionally manipulate the transaction to their advantage, whether to benefit jointly or individually. Achieving integrity, maintaining all transaction properties, and effectively mitigating potential attacks pose significant challenges in such environments.

The offline systems heavily rely on robust encryption technology both in hardware and software. Cryptography plays a crucial role in safeguarding transactions and communications in both digital and physical payment systems. Cryptography guarantees the confidentiality and integrity of data exchanged between transaction parties, facilitates the authentication of their identities, and ensures non-repudiation, preventing any party from denying their involvement in the transaction. Among the key cryptographic frameworks is the Public Key Infrastructure (PKI) [16], where users are assigned a pair of keys. These include a publicly known key called *Public-key* and a privately held key called *Private-key* that remains secret. PKI allows securing communication channels and data, authenticating the identity of communicators, and managing the digital certificates to validate user keys. Two essential cryptographic constructs, digital signatures [17] and digital certificates [18], fortify the security framework. A Digital Signature acts much like a virtual fingerprint, a unique identifier for a person or entity, established using a private key and verifiable via its corresponding public key. This assures that the signatory is genuine and that the signed data are nottampered with from their inception. Analogously, a digital certificate is akin to a digital ID card, binding a public key with its owner’s identity details, such as name and issuing authority, and is critical for affirming identities in electronic transactions. However, the effectiveness of cryptography depends significantly on the secrecy maintained around these cryptographic keys.

Given the risks associated with software-based [19] storage of cryptographic keys and operations, because manipulation is possible if the root key is compromised and also in functional operations like hashing, encryption and decryption, it is advisable to utilize hardware-based security mechanisms [20]. There is a hardware-based approach called a Secure Element (SE) [20] that offers robust protection for storing cryptographic keys. A Secure Element (SE) is a tamper-resistant hardware component designed to securely store sensitive data, such as cryptographic keys, passwords, and personal information, and to perform cryptographic operations in a way that protects these data from unauthorized access or tampering. It is resilient against sophisticated attacks such as ion beams, microchip decapsulation, and scanning microscopes [21]. Besides storage, SEs produce truly random numbers, which eliminates key duplication or theft [22].

**Figure 2:**
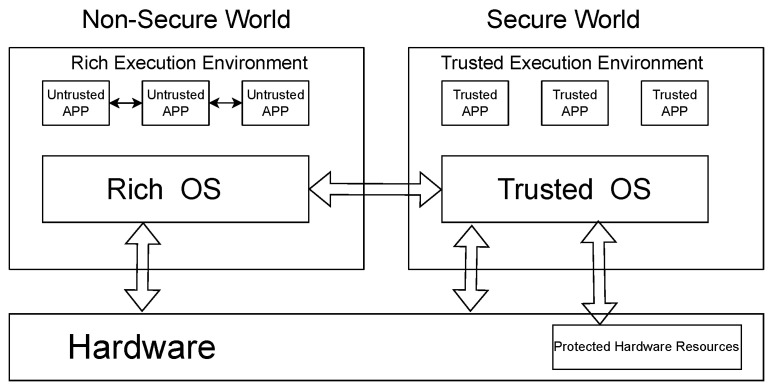
TEE architecture.

Beyond key protection, ensuring the integrity of sensitive payment processes is also crucial. Most, if not all, of the payment solutions have application logic that can be manipulated on the user’s device and poses a severe threat. The Trusted Execution Environment (TEE) [23] provides a solution against this threat. A Trusted Execution Environment (TEE) is a secure area within a main processor. It ensures that the code and data loaded inside it are protected with respect to confidentiality and integrity. The TEE is an isolated mode of operation in which critical code is run and hence cannot be manipulated. This isolation of the TEE makes it immune to theft. For a visual representation of this architecture, refer to Figure 2. The TEE depicted in Figure 2 corresponds to ARM’s architecture. The essence of a Trusted Execution Environment (TEE) is to strategically divide hardware resources, including memory segments, buses, peripherals, and interrupts into two distinct zones: the secure world and the non-secure world. The secure world runs a trusted OS for sensitive operations and houses trusted applications, isolated from the normal world’s Rich Execution Environment (REE) [24] that operates a standard OS and untrusted applications. The communication between these worlds is facilitated through a secure and controlled interface, ensuring data integrity and confidentiality, thus maintaining robust security even if the normal OS is compromised. In summary, the TEE protects sensitive code/data and their execution/storage from threats and attacks emanating from the OS layer [24]. Intel SGX [25], AMD Secure Encrypted Virtualization (SEV) [26], and ARM’s TrustZone [27] are some of the most widely recognized commercial implementations of Trusted Execution Environments (TEEs). In contrast, Keystone [28] stands out as a prominent open-source TEE solution built on the RISC-V architecture, offering a flexible and secure alternative for developers. In the early stages, several vulnerabilities were identified and exploited within TEEs, leading to various security breaches and attacks [5,29,30]. The effectiveness of a Trusted Execution Environment (TEE) relies on the secure loading of a trusted operating system (TOS). A compromised TOS could jeopardize the entire TEE’s security, making it crucial to verify the OS’s integrity before execution. This is where a Trusted Platform Module (TPM) [31] plays a vital role. A TPM is a hardware chip that acts as a root of trust [32]. During the boot process, known as measured boot [33], it takes cryptographic measurements of system components like the TOS. These measurements are stored in Platform Configuration Registers (PCRs) within the TPM [34]. Initially, the Trusted Platform Module (TPM) functions as a hardware root of trust, performing an essential role in the security architecture by first verifying the integrity of the hardware to ensure it has not been tampered with or compromised. Subsequently, the TPM extends this verification process to the firmware, followed by the trusted operating system, and ultimately the rich operating system. Each of these stages is secured through the cryptographic validation of hashes using Platform Configuration Registers (PCRs). This multi-layered approach is foundational in securing the device, as it establishes a robust chain of trust, ensuring the integrity of both hardware and software components. Having a Trusted Platform Module (TPM), Secure Element (SE), and a Trusted Execution Environment (TEE) significantly strengthens the security within the device and processes executed in the device.

Every transaction involves two parties. These parties carry out a transaction using digital devices. For absolute security purposes, it becomes mandatory to establish that every participant and device is trustworthy in the first hand. Imagine if there is a mechanism through which a device can make trustworthy claims about itself with another party. This is what is called *remote attestation*.

Remote attestation verifies the integrity and trustworthiness of a remote system (attester or prover) to another party (verifier). It confirms that the remote system is running the expected software in a secure and unmodified state.

Let us see the mechanism:

1. The attester generates a report containing measurements of its hardware and software configuration (boot sequence, OS, running applications, etc.).
2. This report is cryptographically signed to ensure its authenticity and integrity.
3. The attester sends the report to the verifier.
4. The verifier checks the signature and compares the measurements to known good reference values.
5. If there is a match, the verifier gains trust in the system’s state.

Thus, the above process lets us conclude if the prover’s device can be trusted for secure transactions.

**Figure 3:**
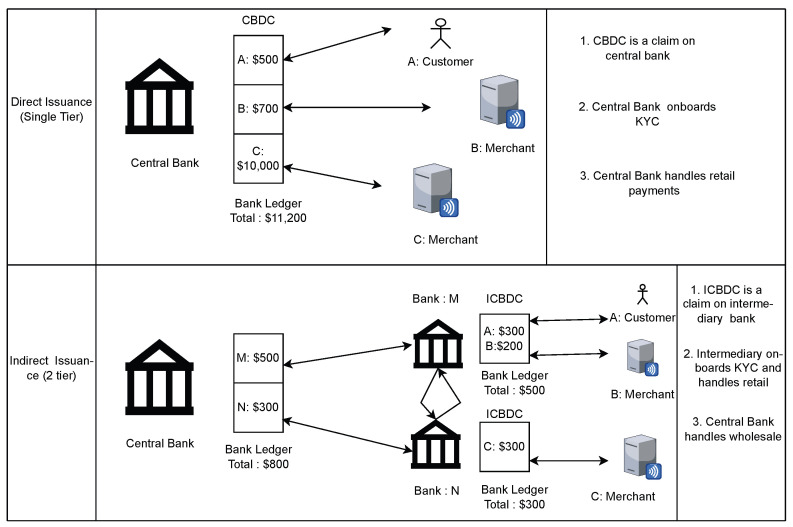
Architecture of CBDC one-tier and two-tier systems.

Let us try to understand Central Bank Digital Currencies (CBDC) [4]. There are two different implementations for CBDCs. The first is a direct, one-tier system where the central bank is the sole manager and distributor of currency to the people. The second and more favoured model is the two-tier system, also called retail CBDC [35], where the central bank issues a wholesale CBDC to intermediary banks, which in turn distribute such retail CBDC to the populace. This model has intermediary banks, which are pivotal in fostering and stabilizing the economy, making use of their sophisticated infrastructure. Figure 3 shows these architectures.

In the discussion of CBDC banking models, the two-tier model stands out for its widespread acceptance and implementation within the financial sector. Although the technical underpinnings of Central Bank Digital Currencies (CBDCs) differ significantly, from a consumer perspective, the process resembles traditional online financial transactions.

There are five security requirements [9] to be met by a CBDC so that it qualifies as a robust payment system: *prevention of double-spending*, *unforgeability*, *non-repudiation*, *verifiability*, and *maintaining anonymity*. No double-spending ensures that digital currencies cannot be illicitly replicated and spent multiple times. Unforgeability protects against the creation of counterfeit currency or the impersonation of entities within the system. Anonymity is a feature that offline payment systems should have; also, providing complete anonymity in CBDCs can cause the same money laundering, tax evasion, and illegal activity problems that happen in conventional financial systems. So, ‘pseudonymity’ [36] is a suitable thing to consider. Our proposed ElasticPay solution is designed to seamlessly integrate with both Central Bank Digital Currencies (CBDCs) and existing digital fiat currency (DFC) systems. Since the transaction flow for both CBDC and DFC is fundamentally similar in the online environment, our approach is adaptable to both systems. For the remainder of this paper, we will refer to both systems collectively as “online banking transactions”, emphasizing their shared digital nature and the fact that our solution caters to both.

## 3. Our Proposed Solution

In addressing security against the above attacks within the proposed offline digital payment system, we propose a synergic combination of technologies—Trusted Platform Module (TPM) [31], Trusted Execution Environment (TEE), and Secure Element (SE). As attacks can be devised with device bootup, compromising operations in the processor and key theft, the proposed trio combination mitigates potential attacks during these phases.

This integrated approach ensures that only authorized applications can access their designated data, effectively shielding them against potential security breaches, even those that might involve sophisticated hardware-based attacks. Moving beyond software-only security solutions is essential due to their limitations in countering advanced cyber threats, highlighting the need for substantial hardware security mechanisms.

The comprehensive security strategy extends to the entire device, securing every interaction and transaction. The TPM, TEE, and SE together create a robust defense system, essential for maintaining data integrity and confidentiality throughout the device’s operation.

The integration of these technologies is increasingly becoming a standard in the smartphone industry. Many new and existing devices on the market are equipped with these features, demonstrating a commitment to high security standards and providing users with the confidence to safely engage in digital transactions.

While the TPM, SE, and TEE can operate independently, their combined use can enhance the security of a device significantly. In our analysis, the concept of ‘trust’ within a device spans its operating system, applications and physical hardware, all of which are expected to meet strict security protocols. A device featuring a Trusted Execution Environment (TEE), Secure Element (SE), and Trusted Platform Module (TPM) clearly demonstrates such trustworthiness.

**Figure 4:**
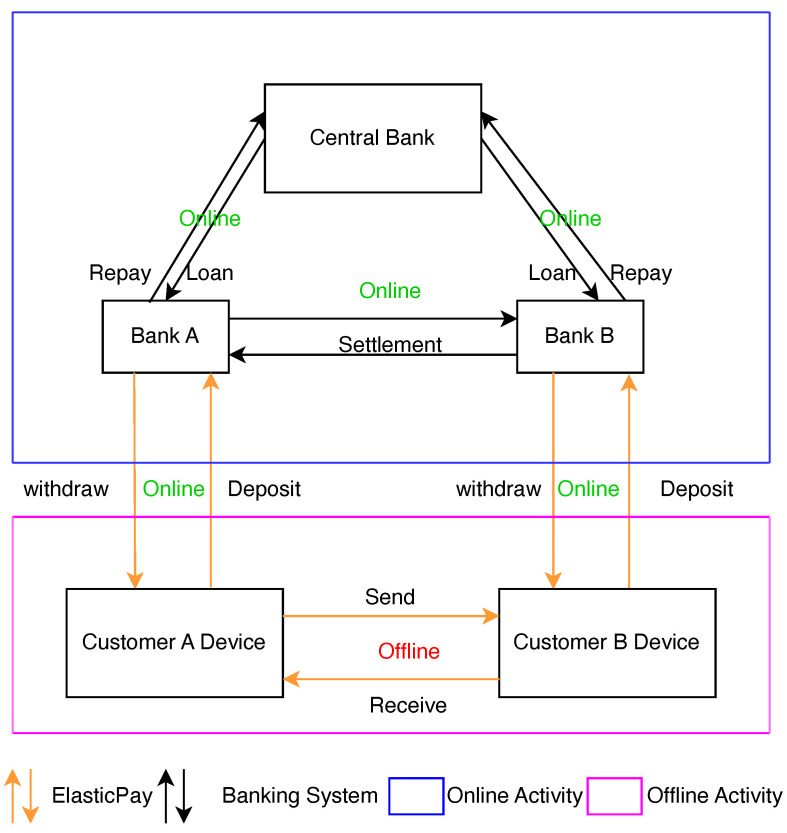
ElasticPay.

Figure 4 illustrates the architectural integration of our proposed ElasticPay solution within the existing online banking transaction framework (Figure 1). ElasticPay extends the capabilities of traditional online banking by introducing a secure offline payment layer, enabling seamless transactions even in the absence of network connectivity, thereby improving accessibility and reliability for users.

Recall that in the preliminaries, the concept of attestation is briefly explained. To prove that we have an integral system with all three features enabled, we employ the attestation technique. In the following, the attestation process that ensures the integrity of ElasticPay is summarized:

- **TPM-based Attestation**: Ensuring the integrity of the device’s boot process and the operating system.
- **TEE-based Attestation**: Verifying that the payment application running within the TEE has not been tampered with and is executing as expected.
- **SE-based Attestation**: Demonstrating that the cryptographic keys have been generated and are stored securely and that any cryptographic operations are performed in a secure environment.

The above attestation verifies that:

- The device has booted with a verified and untampered operating system with the help of the TPM.
- The payment application running is the authentic version and is operating securely within the TEE.
- The cryptographic keys, which are fundamental to the security of the transaction, are generated and stored securely within the SE.

So, whenever we say attestation from here onward, we mean the combination of those three results.

ElasticPay employs NIST P-256 elliptic curve cryptography (ECC) for encryption, coupled with SHA-256 for generating the message digest. We use interchangeably user_A_, Alice (A), and Sender, which all mean the same, and similarly ${\mathrm{user}}_{B}$, Bob(B), and Receiver are the same. Here, we use ‘BS’ for the bank server, which takes care of online registration, third-party verifiers, and root certificate authority. T is a trusted application that runs on the user TEE side, UT is an untrusted application that runs in the normal world, and the entire combined device is called the secure device (SD).

Table 1 and Table 2 provide the notation and terminology used throughout this paper to describe the protocols and algorithms that realize ElasticPay.

ElasticPay comprises three core protocols that facilitate secure and efficient offline digital payments:

1. **Registration Protocol**: This protocol enables the registration of users and their devices within the ElasticPay system. During registration, the Trusted Application (T) on the user’s device establishes its trust through remote attestation and obtains the necessary credentials for secure transaction processing.
2. **Funds Transfer Protocol**: These protocols encompass two distinct scenarios:
  - **Withdraw scenario**: This protocol allows users to securely transfer funds from their online accounts held on the bank server (BS) to their offline wallets managed by the T.
  - **Deposit scenario**: This protocol facilitates the reverse process, enabling users to deposit offline funds from their T wallets back to their online accounts on the BS.
3. **Peer-to-Peer (P2P) Protocol**: This protocol allows for direct transactions between users’ devices. It utilizes cryptographic mechanisms to ensure secure and verifiable transfers even in the absence of network connectivity.

### 3.1. Registration Protocol

The first and crucial step before engaging in any offline transaction is to register with the bank server (BS). This registration is essential as it establishes the authenticity of both the user and their device, which is necessary for gaining the trust of the transaction receiver.

**Figure 5:**
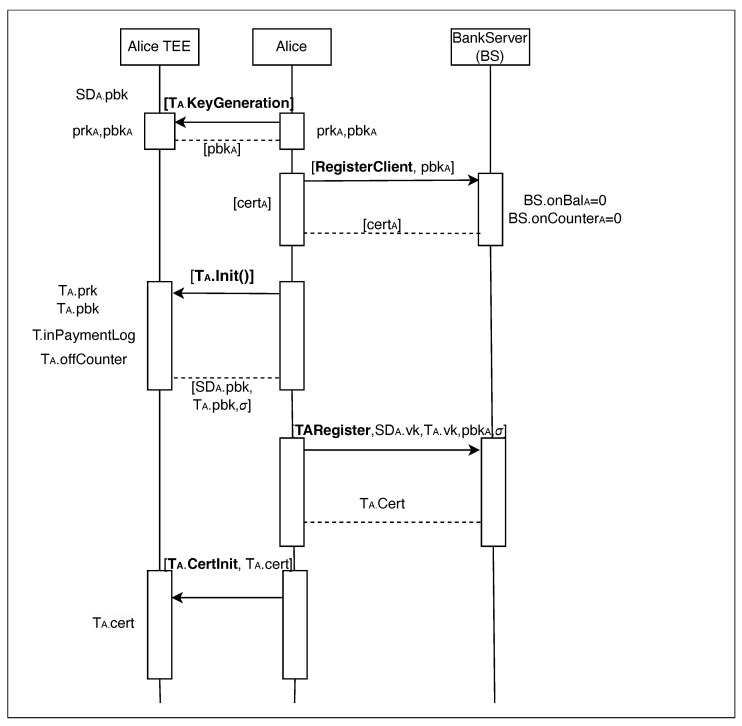
Client registration figure.

The procedure is meticulously documented in a sequence diagram in Figure 5, which clarifies the functions executed within the Trusted Execution Environment (TEE) and those processed on the server side. Although the diagram includes entities like Alice, Alice TEE, and the bank server as separate units, this separation is intended to facilitate a clearer understanding of the distinct roles each plays in the registration process.

Initial user registration within ElasticPay requires an existing online bank account. As illustrated in Figure 5, the user instructs their device’s Trusted Execution Environment (TEE), leveraging the Secure Element (SE) for secure random number generation, to generate a cryptographic key pair, denoted as (${\mathrm{prk}}_{A}$, ${\mathrm{pbk}}_{A}$), using the $T_{A}$. KeyGeneration function. The public key, ${\mathrm{pbk}}_{A}$, is then transmitted to the bank server (BS) for registration. If the BS detects any conflicts, such as the key already being in use, the user is prompted to regenerate and resubmit a new key.

Upon successful registration of the public key, the BS initializes the user’s online balance BS.${\mathrm{onBal}}_{A}$ and online transaction counter Bs.${\mathrm{onCounter}}_{A}$ to 0. The BS then issues a digital certificate ${\mathrm{cert}}_{A}$ to authenticate the user’s account, transmitting this certificate to the user.

The user has now successfully registered his or her online account. For a detailed view of the algorithmic operation that facilitated this registration, please refer to Algorithm 1 in the accompanying documentation.

**Algorithm 1** Client registration protocol.
Alice generates a key pair (${\mathrm{prk}}_{\mathrm{BS}},{\mathrm{pbk}}_{\mathrm{BS}}$$)\leftarrow \mathrm{KeyGen}\left(1^{\lambda }\right)$.C sends **[RegisterClient,** ${\mathrm{pbk}}_{A}$ **]** to BS.Upon receiving **[RegisterClient,** ${\mathrm{pbk}}_{A}$], BS performs the following steps:
(a)Abort if ${\mathrm{pbk}}_{A},\cdot )\in$ BS.Registry.(b)Add (${\mathrm{pbk}}_{A},\mathrm{NULL})$ to BS.Registry.(c)Initialize Alice’s account balance to zero: BS.onBal←A0.(d)Create a certificate ${\mathrm{cert}}_{A}$ such that ${\mathrm{cert}}_{A}$.pbk ← ${\mathrm{pbk}}_{A}$ and ${\mathrm{cert}}_{A}$.sig ← Sign(${\mathrm{pbk}}_{A}$, ${\mathrm{prk}}_{\mathrm{BS}}$), where ${\mathrm{prk}}_{\mathrm{BS}}$ is BS’s private signing key.(e)Send ${\mathrm{cert}}_{A}$ to Alice.


We have seen client registration, and now we will see device registration. The user begins by registering their trusted device via a trusted application, denoted as T, operating within the Trusted Execution Environment (TEE); we denote Alice’s trusted application as $T_{A}$. This process starts with the initiation function $T_{A}$.Init(), which is detailed in Table 3. During this initialization, the application generates cryptographic keys, specifically $T_{A}$.prk (private key) and $T_{A}$.pbk (public key). It also sets initial values: offline balance $T_{A}$.offBal to ‘0’, trusted applications certificate $T_{A}$.cert to undefined (⊥), $T_{A}$.inPaymentLog to undefined (⊥) and transaction counters $T_{A}$.offCounter to ’0’. For clarity and consistency, throughout this paper, the subscript A is used to indicate entities and variables specific to the user Alice. For example, ${\mathrm{pbk}}_{A}$ represents Alice’s public key, $T_{A}$ refers to her trusted application instance and $T_{A}$.KeyGeneration() is the function called by Alice’s T to generate cryptographic keys. Similarly, the subscript B denotes entities and variables associated with another user, Bob.

Following these setups, the application executes a remote attestation mechanism, generating an attestation signature σ and packaging the application’s public key $T_{A}$.pbk. This information is then transmitted to the user’s application layer. Subsequently, the process integrates the secure device’s (SD) root public key ${\mathrm{SD}}_{A}$.pbk with the user’s public key $T_{A}$.pbk and initiates the TARegister protocol with the BS.

The server’s role is to ensure that no other keys are associated with the user’s public key and to validate the attestation signature against known benchmarks. Once verified, the server issues a digital certificate for the user’s trusted application, $T_{A}$.cert. This certificate is sent back to the user. We have previously discussed the importance of ‘trust’ in our system and how remote attestation facilitates this trust. Once remote attestation is completed, the device receives a digital certificate from the certificate authority (CA)—in this context, represented by the BS—confirming the device’s genuineness. ${\mathrm{The T}}_{A}$.cert enables both parties in a transaction, the sender and the receiver, to verify that the device involved has not been tampered with by monitoring changes in the cryptographic hash.

Upon receiving the $T_{A}$.cert from BS, the user stores the certificate within the trusted application residing in the TEE by executing the CertInit operation $T_{A}$.CertInit, which is detailed in Table 3. This step finalizes the secure registration of the trusted application, ensuring that all communications between the parties and transactions performed by $T_{A}$ are authenticated and secure. For a detailed view of the algorithmic operation for device registration, please refer to Algorithm 2.

**Algorithm 2** TA registration protocol.
Alice obtains ($T_{A}$.pbk, $\sigma )\leftarrow T_{A}$.Init() and sends [TARegister, ${\mathrm{SD}}_{A}$.pbk, $T_{A}$.pbk, ${\mathrm{pbk}}_{A}$, σ] to BS.Upon receiving [TARegister, ${\mathrm{SD}}_{A}$.pbk, $T_{A}$.pbk, ${\mathrm{pbk}}_{A}$,σ ] from Alice, Bank server BS does the following steps:
(a)Abort if any of the following conditions is true:
(pbk,⊥)∉A BS.Registry.OEMVerify($T_{A}$.pbk, σ, ${\mathrm{SD}}_{A}$.pbk) ≠ 1.(b)Create a certificate cert such that cert.pbk ← $T_{A}$.pbk; cert.sig ← Sign([$T_{A}$.pbk, ‘T’], ${\mathrm{prk}}_{\mathrm{BS}}$).(c)Send [$T_{A}$.cert] to Alice.(d)Replace (pbk,⊥)A with (${\mathrm{pbk}}_{A}$, cert.pbk) in S.Registry.Upon receiving [$T_{A}$.cert] from BS, client A invokes TA.CertInit($T_{A}$.cert).


### 3.2. Fund Transfer Protocol

**Figure 6:**
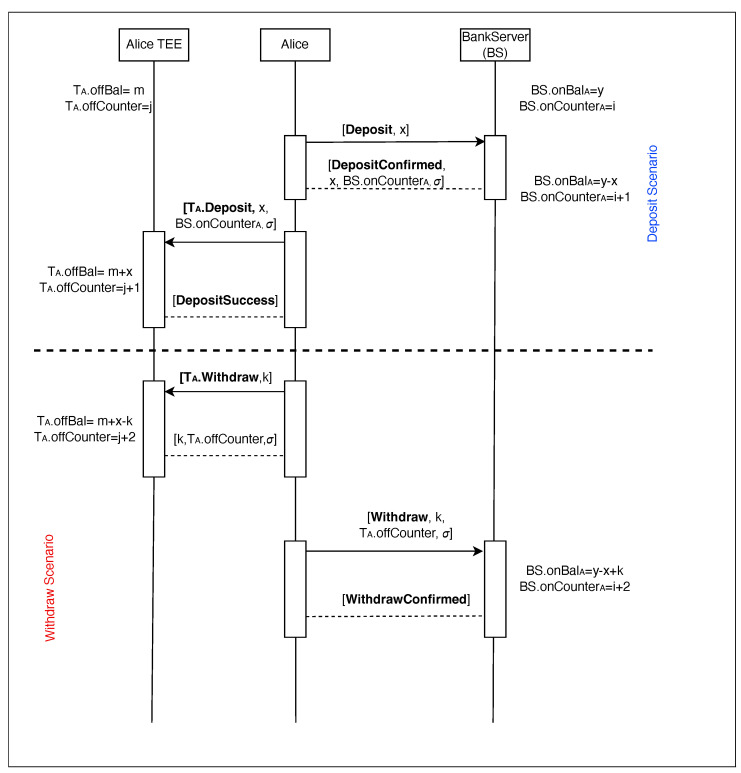
Deposit and withdrawal scenarios between user and bank server.

Having covered the device and user registration process, we now turn our attention to the fund transfer protocol. The sequence diagram for deposit and withdrawal scenarios shown in Figure 6, while the details of the trusted application *T* functions running within the TEE can be found in Table 3.

Let us proceed with the deposit scenario. This crucial procedure allows users to securely transfer funds from their online accounts held on the bank server BS to their device’s offline wallet, facilitating seamless offline transactions.

1. **Deposit Initiation**: To initiate a deposit, the user Alice sends a request to the bank server S specifying the desired amount x to be transferred from her online account to her offline wallet. This request is denoted as [Deposit, x].
2. **Server-Side Validation and Signature**: Upon receiving the deposit request, the BS first verifies that Alice’s online balance BS.${\mathrm{onBal}}_{A}$ is sufficient to cover the requested amount x. If the balance is adequate, the BS debits the amount from her online account BS.${\mathrm{onBal}}_{A}$← BS.${\mathrm{onBal}}_{A}$− x and increments its online transaction counter BS.onCounter←A BS.${\mathrm{onCounter}}_{A}$ + 1. The BS then generates a digital signature σ on the transaction data—which includes the recipient’s public key $T_{A}$.pbk, the amount x, and the updated counter BS.${\mathrm{onCounter}}_{A}$—using its private key BS.prk. Finally, the BS sends a confirmation message *DepositConfirmed* containing the amount, transaction counter, and signature back to Alice.
3. **Trusted Application Verification and Credit**: Alice’s device receives the *DepositConfirmed* message and forwards it to the trusted application T residing in the TEE. The T then executes the $T_{A}$. Deposit function can be found in Table 3, which performs a series of security checks:
  - Verifies that a valid certificate $T_{A}$.cert is present.
  - Confirms that the online transaction counter BS.${\mathrm{onCounter}}_{A}$ matches the expected next value in the T’s offline counter.
  - Validates the authenticity of the signature σ using the BS’s public key ${\mathrm{pbk}}_{\mathrm{BS}}$. If any of these checks fail, the transaction is aborted. Otherwise, the T credits the amount x to Alice’s offline wallet T.offBal ← $T_{A}$.offBal + x and increments its offline counter $T_{A}$.offCounter ← $T_{A}$.offCounter + 1. The T then notifies Alice of the successful deposit with a *DepositSuccess* message.

The deposit scenario, visualized in Figure 6 and formally described in Algorithm 3, enables the secure conversion of online funds into offline funds within the ElasticPay framework. This process involves crucial steps on both the bank server and the user’s trusted device, ensuring the integrity and authenticity of the transaction. This procedure is also called *online fund* to *offline fund* conversion.

**Algorithm 3** Deposit protocol.
Alice sends [Deposit, x] to BS.Upon receiving [Deposit, x] from Alice, server BS does the following steps:
(a)Abort if x > BS.${\mathrm{onBal}}_{A}$.(b)BS.onbal←A BS.${\mathrm{onBal}}_{A}$ - x.(c)BS.i←A BS.$i_{A}$ + 1.(d)Send [*DepositConfirmed*, x, BS.i,Aσ] to A, where σ← Sign([$T_{A}$.pbk, x, BS.${\mathrm{onCounter}}_{A}$], BS.prk).Upon receiving [*DepositConfirmed*, x, BS.onCounter,Aσ] from BS. A invokes T.Deposit(x, BS.onCounter,Aσ).


The incrementing counter mechanism plays a crucial role in preventing replay attacks. In a replay attack, a malicious actor would attempt to fraudulently credit their offline wallet multiple times by repeatedly sending the same, previously valid [Deposit, x, BS.onCounter,Aσ] message to the trusted application T. However, the T maintains its own counter $T_{A}$.offCounter, which is synchronized with the bank server’s counter BS.${\mathrm{onCounter}}_{A}$ during the initial deposit. Subsequent deposit messages are only accepted if the server’s counter is exactly one increment higher than the T’s stored counter. This ensures that each deposit message is unique and can only be processed once, effectively mitigating the threat of replay attacks.

In addition to deposits, ElasticPay enables the secure transfer of funds from a user’s offline wallet back to their online bank account. This is accomplished through the withdrawal protocol, which, as shown in Figure 6, involves a series of interactions between the user’s device and the bank server (BS) and formally described in Algorithm 4.

1. **Withdrawal Initiation and Preparation (Trusted Application)**:
  - The user initiates a withdrawal request through the untrusted application UA, specifying the amount *k* to be transferred from their offline wallet to their online account.
  - The UA communicates this request to the trusted application T by invoking the $T_{A}$.Withdraw function (Table 3).
  - The T first verifies that the user’s offline balance $T_{A}$.offBal is sufficient to cover the withdrawal amount k and that a valid certificate $T_{A}$.cert is present.
  - Upon successful verification, the T debits the amount k from the offline balance $T_{A}$.offBal ← $T_{A}$.offBal − k, increments its internal transaction counter $T_{A}$.offCounter, and generates a digital signature σ over the withdrawal details using its private key $T_{A}$.prk.
  - The TA sends the withdrawal request, including the amount, transaction counter, and signature, back to the UA.
2. **Withdrawal Request and Verification (Bank Server)**:
  - The UA forwards the withdrawal request [Withdraw, k, $T_{A}$.offCounter, σ] to the BS.
  - The BS validates the request by verifying the signature using the T’s public key $T_{A}$.pbk and ensuring the transaction counter $T_{A}$.offCounter is consistent with the expected value on the server side BS.${\mathrm{onCounter}}_{A}$ + 1.
  - If any of the checks fail, the BS aborts the transaction.
  - If the withdrawal request is valid, the BS credits the amount k to the user’s online account BS.onBal←A BS.${\mathrm{onBal}}_{A}$ + k.
  - The BS increments its own transaction counter BS.onCounter←A BS.${\mathrm{onCounter}}_{A}$ + 1.
  - The BS sends a confirmation message *WithdrawConfirmed* to the user, signifying the successful completion of the withdrawal.

The complete details of the withdrawal protocol, including the algorithm executed by the *T* and the interaction between entities, are outlined in Algorithm 4 and illustrated in Figure 6.

**Algorithm 4** Withdrawal protocol.
Client A sends [Withdraw, k, $T_{A}$.offCounter, σ] to Bank-server (BS), where [k, $T_{A}$.offCounter, σ] ← $T_{A}$.Withdraw(k).Upon receiving [Withdraw, k, $T_{A}$.offCounter, σ] from A, BS performs the following steps:
(a)Abort if $T_{A}$.offCounter ≠ BS.${\mathrm{onCounter}}_{A}$ + 1 or SigVerify([k, $T_{A}$.offCounter], σ, $T_{A}$.pbk) ≠ 1.(b)BS.onBal←A BS.${\mathrm{onBal}}_{A}$ + k.(c)BS.onCounter←A BS.${\mathrm{onCounter}}_{A}$ + 1.(d)Send [*WithdrawConfirmed*] to A.



### 3.3. Peer-to-Peer (P2P) Protocol

**Figure 7:**
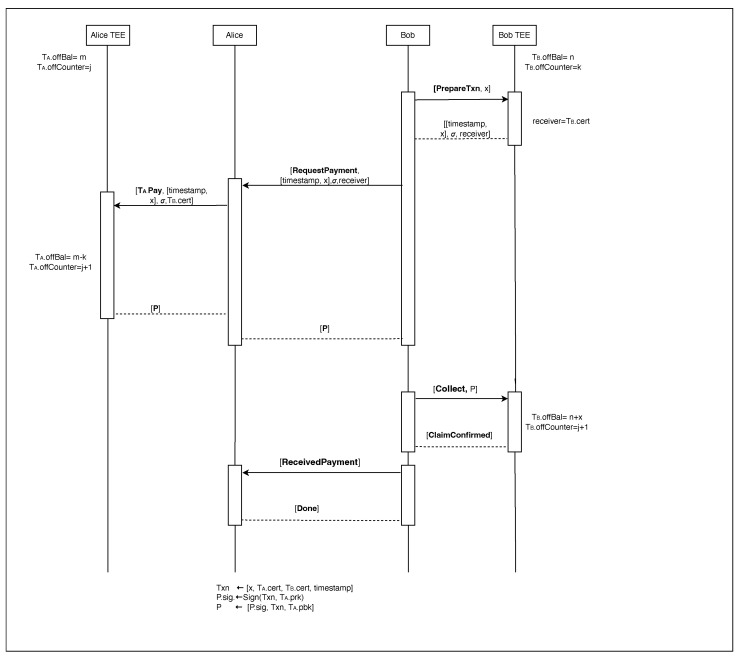
Interaction between peers.

The P2P protocol facilitates direct offline transactions between two users, as illustrated in Figure 7. All operations within the Trusted Execution Environment (TEE) are detailed in Table 3, while the complete P2P protocol is outlined in Algorithm 5.

1. **Transaction Preparation (Receiver):**
  - The receiver (Bob) initiates the transaction by instructing his trusted application $T_{B}$ to prepare a transaction for amount x, using the PrepareTxn (x) function.
  - $T_{B}$ retrieves a timestamp from the device’s clock and generates a signature σ on the amount x and timestamp using its private key $T_{B}$.prk.
  - Bob sends the signed transaction request [x, timestamp, $T_{B}$.cert, σ] to the sender Alice.
2. **Payment Initiation and Verification (Sender):**
  - Alice’s untrusted application UA receives the signed transaction request and forwards it to the sender’s trusted application $T_{A}$ by invoking the $T_{A}$.Pay function.
  - $T_{A}$ verifies:
    – The validity of Bob’s signature using his public key $T_{B}$.cert.pbk.
    The presence of a valid certificate $T_{A}$.cert.
    The sufficiency of funds in Alice’s offline wallet $T_{A}$.offBal ≥ x.
  - If all checks pass, $T_{A}$ debits the amount x from Alice’s offline balance $T_{A}$.offBal and increments its transaction counter $T_{A}$.offCounter by one.
  - It creates the transaction payment P with the following information P.amount ← x, P.sender ← $T_{A}$.cert, P.receiver ← $T_{B}$.cert, P.timestamp ← timestamp, Txn ← [P.amount, P.sender, P.receiver, P.timestamp], P.sig ← Sign(Txn,$T_{A}$.prk).
  - Sends P to the receiver (Bob) where P ← [Txn, P.sig, $T_{A}$.pbk].
  - $T_{A}$ returns P to the UA, which transmits it to Bob.
3. **Payment Collection (Receiver):**
  - Bob’s UA receives the payment object P and forwards it to $T_{B}$.
  - $T_{B}$ performs verification using the PayVerify function (Algorithm 6):
    – Validates Alice’s certificate $T_{A}$.cert.
    Verifies the signature on P using Alice’s public key $T_{A}$.pbk.
    Ensures P is not a duplicate transaction P ∉ $T_{B}$.inPaymentLog.

**Algorithm 5** Peer-to-peer protocol.
B sets receiver ← $T_{B}$.cert if $T_{B}$.cert ≠⊥ and calls $T_{B}$.PrepareTxn in his trusted application T. Otherwise, receiver ← ${\mathrm{cert}}_{B}$ and calls prepare transaction Algorithm 7 from his untrusted application UT. The output is forwarded to B and then forwarded to A with message including message ‘*RequestPayment*’ [*RequestPayment*, [timestamp, x], σ, receiver].Upon receiving [*RequestPayment*, [timestamp, x], σ, receiver] from B, client A sends P to B where P ← TA.Pay (x, timestamp, receiver).Upon receiving P from A, client B performs the following steps:
(a)Abort if any of the following conditions is true: PayVerify(P) ≠ 1 or P.receiver ≠ receiver or P ∈ B.inPaymentLog.(b)B adds P to B.inPaymentLog in his UT and sends [*ReceivedPayment*] to A.(c)If P.receiver.type = “T”, then B calls $T_{B}$.Collect (P).(d)Otherwise, B engages in the Claim protocol Algorithm 8 with BS as soon as B is online.

- If all checks succeed:
  – If Bob provided his online banking certificate ${\mathrm{Cert}}_{B}$, UA initiates the Claim protocol (Algorithm 8) to eventually credit his online account.
  If Bob provided his T certificate $T_{B}$.cert, UA initiates the $T_{B}$.Collect protocol to eventaully credit his offline account.

**Algorithm 6** PayVerify function.PayVerify(P):Return 1 if and only if all of the following conditions hold:
TACertVerify(P.sender) = 1 andSigVerify([P.amount, P.sender, P.receiver, P.timestamp], P.sig, P.sender.pbk) = 1.


**Algorithm 7** PrepareTxn.
PrepareTxn (x):If ${\mathrm{cert}}_{B}$ = ⊥ then abort.receiver ← ${\mathrm{cert}}_{B}$.Output [[timestamp, x], σ, receiver] where σ← Sign([timestamp, x], ${\mathrm{prk}}_{B}$)


**Algorithm 8** Claim protocol.
B sends [Claim, P] to BS.Upon receiving [Claim, P] from B. Bank server BS performs the following steps.
(a)Abort if any of the following conditions is true:
P.receiver.type ≠ ‘UA’.PayVerify(P) ≠ 1.P ∈ BS.paymentLog
(b)BS.onBal ← BS.onBal + P.amount.(c)Add P to BS.inpaymentLog;(d)Send [*ClaimConfirmed*] to B.



### 3.4. Integration of ElasticPay with Digital Fiat Currencies

ElasticPay can seamlessly integrate with digital fiat currency systems, much like its integration with Central Bank Digital Currencies (CBDCs), as illustrated in the sequence diagram in Figure 5 and detailed in Section 3.1. In this scenario, intermediary banks, rather than central banks, handle Know-Your-Customer (KYC) processes and customer verification. When a user (the sender) converts their online balance to offline, their corresponding intermediary bank, Bank ‘A’, manages the process. This includes setting up the sender’s device, remote attestation, and certificate assignment.

For offline transactions, the receiver’s device, managed by their intermediary bank, Bank ‘B’, verifies the sender’s certificate issued by Bank ‘A’. This verification involves a two-layer process: first, validating the user’s certificate issued by Bank ‘A’, and then ensuring that Bank ‘A’s public key is listed in the receiver’s Trusted Execution Environment (TEE). This dual-layer verification ensures the authenticity and security of the transaction, allowing only authorized banks to validate certificates.

This integration method applies the same ElasticPay protocols to both CBDCs and digital fiat currencies, ensuring seamless compatibility across different systems.

## 4. Experimentation

The primary objective of our research was to develop ElasticPay. This system aims to significantly reduce the attack surface commonly associated with digital transactions. To achieve this level of security, we utilized Trusted Execution Environments (TEEs), Trusted Platform Modules (TPM-like features) and Secure Elements (SEs), which are essential for maintaining the integrity and confidentiality of transactions.

As part of our proposed approach, we utilize Trusted Execution Environments (TEEs), Trusted Platform Modules (TPMs), and Secure Elements (SEs) to enhance security. These components typically operate collaboratively to fortify security measures. However, in Apple devices, these functionalities are consolidated into a single hardware component in the system on a chip (SoC) known as the Secure Enclave. This integration provides robust isolation from the main processor and system, ensuring that all sensitive operations are securely managed and segregated from the rest of the device architecture. Today, most mid-range mobile devices are equipped with technologies such as TPM, TEE, and SE, either separately or as part of the SoC, as their inclusion has become a mandatory standard. These technologies play a vital role in enhancing device functionality and improving the overall user experience.

### 4.1. Why iPhone?

**Visualizing Apple’s Security Architecture**

**Figure 8:**
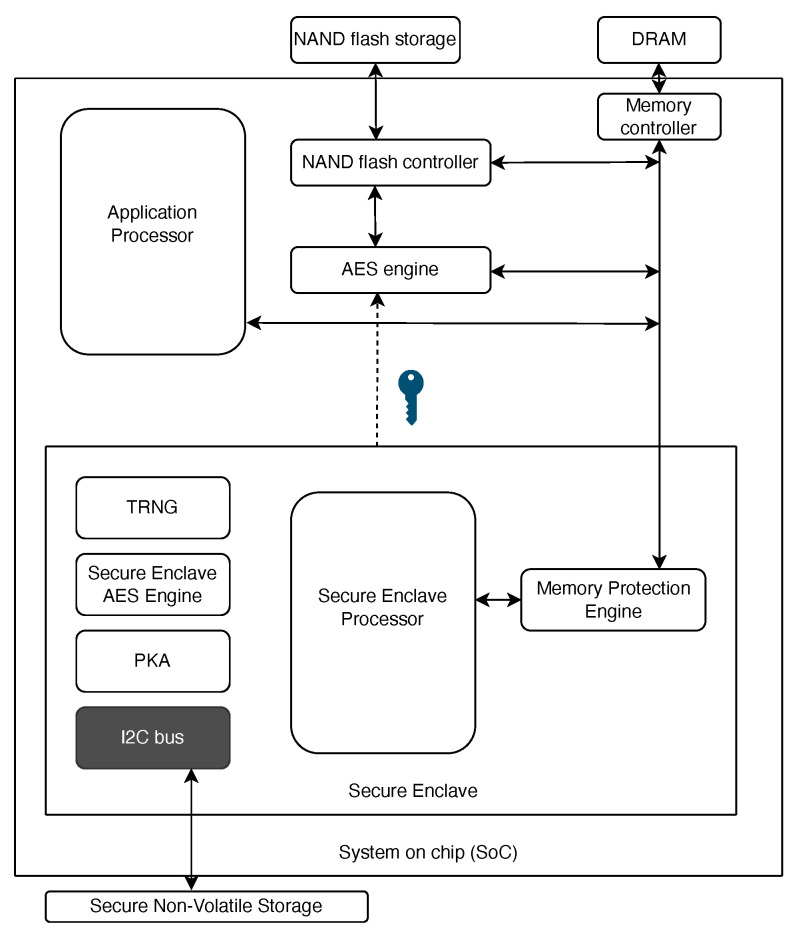
Apple SoC.

Apple’s Secure Enclave is a comprehensive security system within its devices that employs a dedicated Secure Enclave Processor for isolated operations, a Memory Protection Engine for encrypted storage, and a Secure Enclave Boot Monitor for secure booting. It also includes a True Random Number Generator for cryptographic operations, Root Cryptographic Keys unique to each device, an AES Engine for hardware-accelerated encryption, and a Public Key Accelerator for secure communications. Additionally, secure non-volatile storage safeguards sensitive data, all combining to create a robust security infrastructure for Apple devices. Please refer to Figure 8 for a depiction of the Apple SoC architecture. For an in-depth understanding, you can refer to [37].

The iPhone was chosen as our primary experimental platform because of its sophisticated and integrated security architecture. This architecture includes several key features.

Secure Boot and Measured Boot: The iPhone’s boot process involves several stages of security checks—from the immutable Boot ROM, through the Low-Level Bootloader (LLB), to the iBoot that loads the iOS kernel. Each stage ensures that only Apple-signed software can proceed, effectively preventing unauthorized modifications.

Secure Enclave: Integral to Apple’s hardware, the Secure Enclave provides an isolated execution environment similar to a TEE. It is designed for secure cryptographic operations and sensitive data handling, effectively protecting cryptographic keys used during transactions and other operations.

Apple Keychain: We leveraged Apple Keychain for securely storing sensitive transaction data such as account balances, digital certificates, and transaction counters. Keychain’s robust encryption features and anti-replay mechanisms make it an ideal tool for securing financial transactions.

App Sandboxing: App sandboxing is a crucial security feature of iOS that confines each app to its own secure environment, preventing it from accessing data or resources belonging to other apps without explicit user permission. In our experiment, app sandboxing plays a vital role in isolating the peer-to-peer offline payment application, shielding it from potential threats and ensuring that sensitive payment data remain confidential and secure.

Memory Manipulation, Memory Dump, and Sniffing Prevention: The iPhone incorporates advanced security mechanisms, such as Data Execution Prevention (DEP) and Address Space Layout Randomization (ASLR), to prevent memory manipulation, memory dump attacks, and data sniffing. These defences safeguard the application’s data from unauthorized access and manipulation, ensuring the integrity and confidentiality of payment transactions.

Isolated Application Execution and Trusted Execution Environment (TEE): The iPhone’s security model ensures the isolated execution of applications, preventing unauthorized access or manipulation of data between applications. The Trusted Execution Environment (TEE), represented by the Secure Enclave, provides a secure and isolated environment for executing sensitive operations, such as cryptographic operations and biometric authentication. This enhances the security of the offline payment system by shielding sensitive operations from potential threats and vulnerabilities.

Trusted Operating System Loading: During the boot process, the iPhone verifies the integrity of the operating system using cryptographic signatures and secure boot mechanisms. This ensures that only a trusted operating system, signed by Apple, is loaded into the device’s memory. Additionally, hardware-based security features, such as the Secure Enclave, monitor and enforce the integrity of the operating system, preventing unauthorized modifications or tampering.

Secure Communications: iOS implements strong encryption protocols for network communications, ensuring that data transmitted between your application and external servers are encrypted and secure. This protects against eavesdropping and data interception, maintaining the confidentiality of sensitive information exchanged during transactions.

By leveraging these advanced security features of the iPhone, our experiment establishes a Trusted Execution Environment for the offline payment system, ensuring the integrity and confidentiality of payment transactions. These robust security measures protect against potential threats and vulnerabilities, safeguarding the broader economic ecosystem from security risks and ensuring user privacy.

### 4.2. ElasticPay Implementation

**Figure 9:**
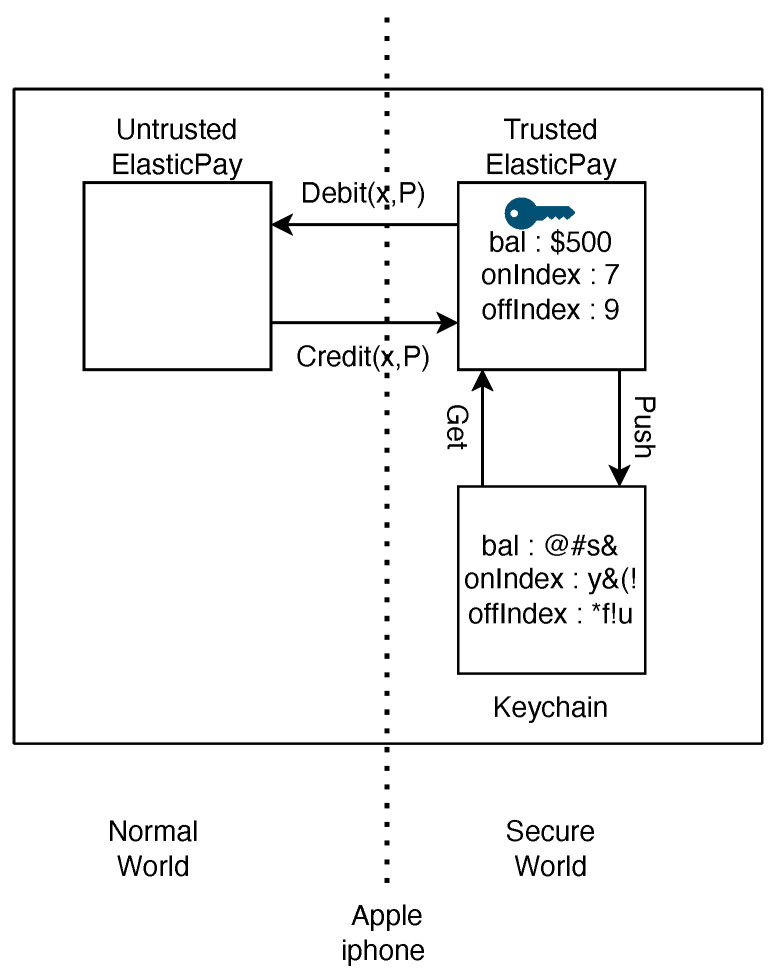
ElasticPay overview in Apple iPhone.

In ElasticPay, transaction processing is handled within the Secure Enclave to ensure the integrity and confidentiality of each transaction. Interaction with the Apple Keychain allows for the secure retrieval and storage of encrypted data as shown in Figure 9. During a transaction, encrypted data are fetched from the Keychain, decrypted within the Secure Enclave, and updated and re-encrypted before being securely stored back. This process ensures that sensitive transaction data are protected throughout their lifecycle. With our system, users can initiate credit or debit operations by directly interacting with the trusted ElasticPay service. To do so, they will use the regular (non-trusted) ElasticPay application and provide the necessary transaction details. The application gathers the inputs and securely relays them to the trusted service. After processing is complete, ElasticPay provides results that the user can then independently forward to the appropriate party—such as a bank server or the transaction recipient.

In the initial section of our discussion, we covered the standard remote attestation mechanisms involving the TEE, TPM and SE. Moving to the experimental phase with an Apple iPhone, it is noteworthy that Apple employs a robust code-signing process to verify the authenticity of applications before they run. Integrated with this, the Secure Enclave validates the software’s integrity by confirming firmware and booting protocols and by generating detailed attestation reports. This comprehensive validation process effectively prevents any unauthorized or altered applications from executing, reinforcing Apple’s strong commitment to security.

### 4.3. Comparison with Android Devices

While our focus was on the iPhone due to its advanced features, it is important to note that similar security components such as TEEs, TPM-like features, and SEs are also present in many Android devices. These components are theoretically sufficient to support secure applications like ElasticPay. However, the additional security layers provided by Apple, including the unified ecosystem and advanced Secure Enclave, offer enhanced protections that are particularly valuable for financial applications demanding stringent security measures.

### 4.4. Summary

The integration of these sophisticated technologies within the iPhone platform provides an ideal testing ground for ElasticPay, demonstrating that leveraging this integrated security architecture enables secure, efficient, and reliable offline transactions.

### 4.5. Experimental Setup

**Figure 10:**
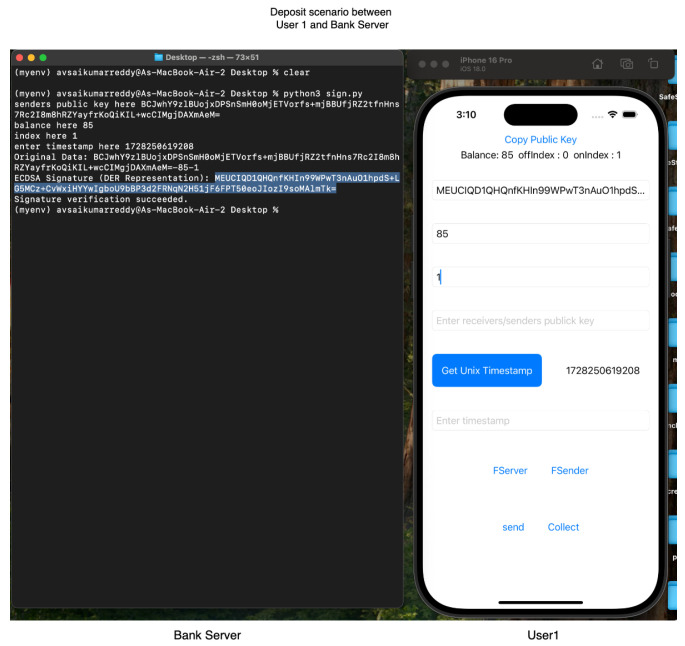
Screenshot from the ElasticPay application illustrating the deposit process, depicting a transfer of 85 units to User1 from the bank server.

**Figure 11:**
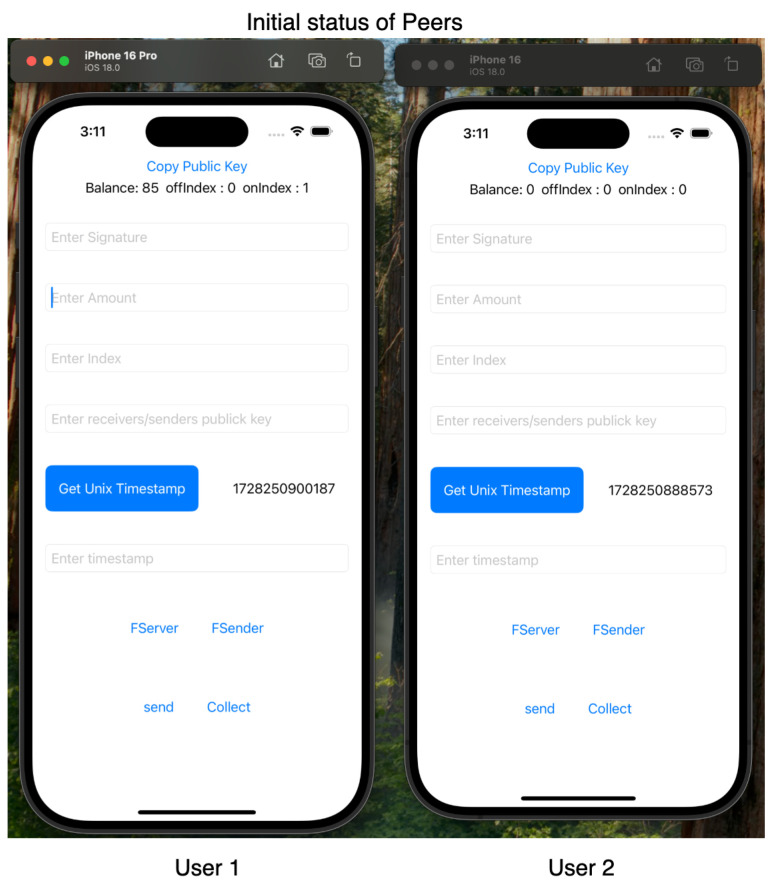
Screenshot of the initial status for User1 and User2 in the ElasticPay application.

**Figure 12:**
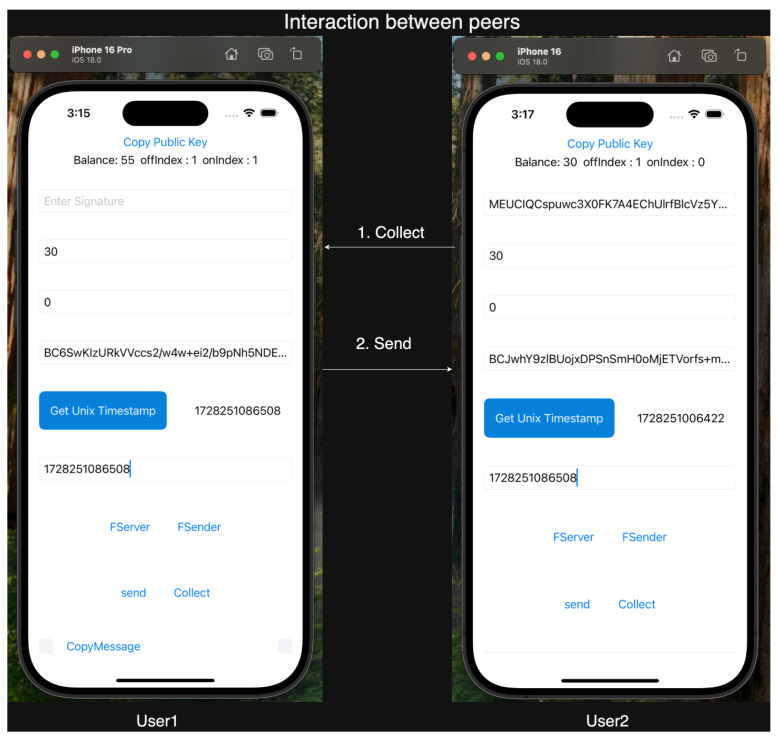
Screenshot of an offline transaction involving a transfer of 30 units between User1 and User2, highlighting the updated balances for both users.

For our experiments, we employed Apple iPhone 16 and iPhone 16 Pro devices, both running iOS 16, to leverage the Multipeer Connectivity framework. Our testing confirmed ElasticPay as a viable solution. Screenshots of the ElastciPay application, implemented according to the established protocols, are shown in Figure 10, Figure 11 and Figure 12.

The Multipeer Connectivity framework facilitates seamless communication by dynamically choosing between Bluetooth Low Energy (BLE) and Peer-to-Peer Wi-Fi. When devices are in close proximity, Bluetooth Low Energy is prioritized due to its low power consumption and efficiency, in line with our commitment to sustainability. In cases where devices are farther apart, the framework defaults to Peer-to-Peer Wi-Fi, which does not require an external Wi-Fi access point and ensures a stable connection over longer distances. Given that the transaction messages exchanged are relatively small, Bluetooth is preferred for communication, offering an optimal balance between speed, power efficiency, and responsiveness. During offline transactions, it is anticipated that devices will be in close range, making Bluetooth the typical communication medium. However, in situations where Bluetooth is unavailable or impractical, Wi-Fi serves as a reliable fallback, ensuring continued functionality.

The latency comparison between ElasticPay and a basic transaction system is presented in Table 4. In a single transaction, the total message size for ElasticPay is 80 bytes, which includes the following components:

- 16 bytes for the message payload (comprising the Unix timestamp and the amount);
- 32 bytes for the SHA-256 hash of the message;
- 32 bytes for the encrypted hash (signature), which is generated using NIST P-256 elliptic curve cryptography (ECC).

At the sender’s side, the system performs encryption of the 16-byte payload, and at the receiver’s side, decryption occurs to verify the authenticity. This process is coupled with hashing (SHA-256) to generate the message digest for signing. In contrast, in a basic transaction, no encryption or decryption is involved. The system only transfers an 8-byte message (usually just the amount), making it much simpler and more efficient in terms of data size and computational overhead.

Table 4 presents a latency comparison between ElasticPay (the ElasticPay implementation) and a basic transaction system, which operates without encryption, decryption, or hashing. Both systems were tested on the same experimental device. Both systems follow a sequential transaction model, where each transaction is processed only after the previous one is successfully completed. This behaviour is due to the lack of a central authority in the offline environment in coordinating or ordering transactions.

## 5. Related Work

In the landscape of smartphone-based offline payment systems, several works stand out, specifically those by [11,12,13] all of which explore different security frameworks. Both [11,13] primarily utilize Trusted Execution Environments (TEEs) to secure transactions. While this method ensures some level of security, it has been noted that relying solely on TEE can expose the system to various attacks, especially if key management practices are not robust [11,13].

Ref. [12] expand on this by combining TEE with Secure Elements (SEs), arguing that this duo enhances security by making it more challenging to extract sensitive cryptographic keys [12]. However, ref. [12] also acknowledges vulnerabilities during the TEE’s boot process, indicating that even this more complex system is not impervious to threats.

We note key approaches from researchers such as [11,12,13]. These works often grapple with privacy compromises; for instance, Refs. [12,13] require senders to share comprehensive transaction histories with recipients to establish trust, a practice that significantly undermines privacy [11] and attempts to mitigate replay attacks by using an index that increments with each transaction. This index ensures that each transaction is unique, preventing participants from reusing transaction data. However, this approach inadvertently reveals the number of previous transactions. As a result, it can indirectly leak sensitive information, such as the fact that a participant has completed ‘n’ number of transactions.

Our proposed solution, ElasticPay, addresses the limitations of previous approaches by integrating Trusted Platform Modules (TPMs) alongside TEEs and SEs. This multi-layered approach not only enhances security but also prioritizes user privacy. Instead of exposing full transaction histories, ElasticPay utilizes unique Unix epoch timestamps to identify transactions. During a transaction, the receiver selects a timestamp and the sender signs the transaction using this timestamp. This method effectively prevents replay attacks, similar to using an index, but offers the added benefit of efficient log(n) search time when verifying the non-existence of a particular transaction within a large set of transactions, as opposed to the linear O(n) search required for random numbers. By minimizing data exposure and ensuring transaction immutability, ElasticPay establishes a robust security framework for offline payments.

Table 5 and accompanying text present a clear, comprehensive view of how our solution leverages existing technologies while addressing their limitations to enhance both security and privacy.

Table 6 summarizes how ElasticPay addresses five key security requirements for Central Bank Digital Currencies (CBDCs): prevention of double-spending, unforgeability, non-repudiation, verifiability, and maintaining anonymity.

## 6. Discussion

Let us see the key features of ElasticPay.

1. **Double Spending Protection**: In ElasticPay, transactions are securely processed on a trusted device, ensuring that balance deductions are safeguarded. A payment token can only be generated through a specific function, effectively limiting the user’s ability to initiate multiple transactions using the same token. The token contains essential details such as the recipient’s name and the amount, which are integral to the transaction. Any attempt to alter the token would disrupt its cryptographic signature, making it invalid and preventing misuse or redirection of funds.
2. **Replay Attack Prevention**: Replay attacks involve the unauthorized reuse of a valid message or token. In the ElasticPay system, both the sender and receiver utilize trusted devices. When the receiver submits a received token to their Trusted Execution Environment (TEE) for processing, the TEE assesses whether the token has previously been utilized. If it detects that the token has already been used, it terminates the operation to prevent duplication. Similarly, during transactions intended to increase an online balance, the bank server (BS) checks for token reuse and will halt the transaction if a replay attempt is detected. This system ensures robust protection against replay attacks.
3. **Forgery Prevention**: Forgery typically manifests in two forms: impersonation using someone else’s identity and the creation of counterfeit currency. In the system described, both forms of forgery are effectively thwarted. To be deemed trustworthy, a user must possess a digital certificate issued by an authoritative entity. This certificate can only be obtained after the user’s identity has been verified by the authority, ensuring that each participant’s identity is authenticated and cannot be falsely assumed or replicated.
4. **Non-Repudiation**: In the transactions, participants are unable to deny their involvement as each transaction is confirmed through the exchange of digital signatures and explicit consent. This ensures that all parties acknowledge and accept the transaction details, securing accountability and traceability.
5. **Side-Channel Attack Prevention**: Throughout the transaction process, our system ensures that no external information is leaked, safeguarding against side-channel attacks. This is achieved by leveraging the isolation features of TEEs and the encryption capabilities of SEs to shield transaction details from being exposed through indirect means such as power consumption or electromagnetic emissions.
6. **Mitigation of Man-in-the-Middle Attacks**: All communications within our system are fully encrypted using advanced cryptographic protocols facilitated by the TPM and SE, effectively neutralizing the threat of man-in-the-middle attacks where attackers could intercept or alter messages.
7. **Hardware Control Attack Resilience**: Even if a device owner attempts to manipulate the hardware to gain unauthorized access to application data, they will be thwarted by comprehensive encryption measures. Data stored on the disk, active in main memory, or being processed remain encrypted, with keys securely managed within the SE. Access to the SE is stringently controlled, making it extremely challenging to extract sensitive information, even with sophisticated laboratory attacks.
8. **Software Control Attack Defense**: Our system is fortified against software tampering through the use of secure boot processes integrated with TPM technology. Before any software, firmware, or operating system loads on the device, it is authenticated for integrity. Any detected tampering causes the boot process to halt, preventing compromised software from executing.
9. **Privacy Preservation in Transactions**: The privacy of users is meticulously preserved; neither party in a transaction needs to disclose sensitive past transaction details or current balance information to establish trust. Only essential information required for the completion of a transaction is shared, minimizing the exposure of private data.
10. **Recovery from Transaction Failures**: In cases where a transaction might freeze or fail—for example, if a “debited” message does not reach the receiver—our system provides mechanisms for retransmission. If retransmission is not possible, the transaction is recorded as failed and the system, upon later review by an authorized entity, can invalidate and regenerate the token. These sensitive operations are handled with utmost care to ensure transaction integrity.
11. **Anonymity and Identity Disclosure**: Cryptocurrency transactions are typically understood as anonymous, yet they more accurately offer pseudonymity. When engaging in these transactions, individuals are represented by public keys, not by their real-world identities. These keys, which act as digital identifiers on the blockchain, do not disclose personal details but can be connected to transaction histories. In the context of Central Bank Digital Currencies (CBDCs), public keys undergo verification and certification by a certificate authority, which underpins each with a confirmed identity. While users can change their public keys to maintain transactional privacy, their identities are still anchored by background checks and KYC (Know Your Customer) compliance managed by the certificate authority. Therefore, the anonymity provided is not absolute; it is conditional and regulated, ensuring that privacy in financial activities is preserved unless a legal intervention necessitates unveiling the identity. This framework of pseudonymity also extends to offline transactions, where similar cryptographic safeguards are employed to protect individual privacy, with mechanisms in place for identity verification under specific circumstances.

The Societal Benefits of ElasticPay.

Our system enhances financial inclusion by enabling transactions without internet access, providing a viable solution for underbanked regions and bridging the gap for millions excluded from digital financial services. Its offline capability ensures transactions can be processed during internet outages or in remote areas, increasing the resilience of the digital payment infrastructure. Designed to be privacy issue-free, it protects user data even offline, addressing concerns about data breaches and unauthorized access, thus making digital payments more secure. Additionally, offline payment methods reduce dependency on expensive internet infrastructure, offering a cost-effective solution for users and service providers. The system’s offline functionality also makes it scalable across various environments, from urban centers to rural areas, without significant infrastructure changes.

## 7. Conclusions and Future Work

Initially, our research focused on comprehending the technologies enabling offline payment transactions and exploring their potential applications. However, we identified crucial security and privacy concerns in existing approaches, prompting the development of ElasticPay. Previous work often lacked in-depth discussions of the underlying hardware components—specifically, how they contribute to security and why they are essential for offline payments. Our contribution lies in a comprehensive analysis of these hardware elements and a simplified yet enhanced approach to offline transactions that prioritizes both security and user privacy. ElasticPay not only presents clear and accessible protocols but also addresses transaction failure scenarios that prior work overlooked.

We recognize the inconvenience caused when a debited amount fails to reach the receiver, potentially leaving the user unable to access those funds while offline. To mitigate this, ElasticPay includes a mechanism for storing failed transactions locally and depositing them back to the bank server once an online connection is re-established. While this solution significantly improves the system’s reliability, we acknowledge that the temporary inaccessibility of funds remains a drawback. In addition to this, another limitation of ElasticPay—shared by all offline payment systems—is the sequential transaction model. In these systems, each transaction must be processed one at a time, which can create bottlenecks, especially in high-traffic business outlets. In contrast, online payment systems typically utilize parallel processing on servers with central authority intervention, allowing multiple transactions to be processed simultaneously. This approach benefits high-volume outlets by enabling faster transaction processing, reducing delays, and improving the overall customer experience.

Both of these limitations—fund inaccessibility during offline transactions and the sequential processing of transactions—highlight areas for future development. To enhance the system’s performance and user experience, we plan to extend ElasticPay by implementing automatic recovery of failed transactions without the need for a central authority, as well as exploring ways to enable parallel transaction processing in offline scenarios.

## Figures and Tables

**Table 1:** Protocol variables and their scope.

Variable	Description	Scope
BS.Registry	Bank server’s registry of valid UTA certificates	BS
BS.${\mathrm{onBal}}_{A}$	Client A’s online balance stored at BS	BS
BS.${\mathrm{onCounter}}_{A}$	Client A’s transaction counter maintained by BS	BS
SD	Client A’s secure device/hardware	A
T	Client A’s trusted application deployed on TEE	A
UT	Client A’s untrusted application deployed on REE	A
(${\mathrm{prk}}_{\mathrm{BS}}$, ${\mathrm{pbk}}_{\mathrm{BS}}$)	Bank server’s signing key pair	(BS, Global)
(${\mathrm{prk}}_{A}$, ${\mathrm{pbk}}_{A}$)	Client A’s signing key pair	(A, Global)
($T_{A}$.prk, $T_{A}$.pbk)	$T_{A}$’s signing key pair	($T_{A}$, Global)
(${\mathrm{SD}}_{A}$.prk, ${\mathrm{SD}}_{A}$.pbk)	Client A’s secure device (SD) root key pair	(SD, Global)
${\mathrm{cert}}_{A}$	Certificate for client A consisting of ${\mathrm{pbk}}_{A}$ and a signature on it by BS certifying that ${\mathrm{pbk}}_{A}$ was issued by BS	Global
$T_{A}$.cert	Certificate for $T_{A}$ consisting of $T_{A}$.pbk and a signature on it by BS certifying that $T_{A}$.pbk was issued by BS	Global
$T_{A}$.i	Client A’s server index maintained by $T_{A}$	$T_{A}$
$T_{A}$.j	Client A’s payment index maintained by $T_{A}$	$T_{A}$
$T_{A}$.offBal	Client’s A’s offline balance	$T_{A}$
$T_{A}$.offCounter	Client’s A’s offline transaction counter	$T_{A}$
$T_{A}$.inPaymentLog	List of offline payments received by $T_{A}$	A, $T_{A}$, BS
P.amount	Amount of money transferred by payment P	Holder of P
P.sender	Certificate of the sender of payment P	Holder of P
P.receiver	Certificate of the receiver of payment P	Holder of P
P.timeStamp	Time stamp when payment P was created	Holder of P
P.type	Type of payment P (“Basic” or “Conditional”)	Holder of P

**Table 2:** Protocol functions and their scope.

Function	Description	Scope
Hash (x)	Outputs a cryptographic hash of x	Global
Sign (x, prk)	Outputs a signature of x signed with private key prk	Global
SigVerify (x, σ, pbk)	Outputs 1 iff signature σ over x using public key pbk is valid	Global
CertVerify (cert, ${\mathrm{pbk}}_{\mathrm{BS}}$)	Outputs 1 iff SigVerify (cert.pbk, cert.σ, ${\mathrm{pbk}}_{\mathrm{BS}}$) $\overset{?}{=}$ 1	Global
TACertVerify (cert, ${\mathrm{pbk}}_{\mathrm{BS}}$)	Outputs 1 iff SigVerify ([cert.pbk, ’TA’], cert.σ, ${\mathrm{pbk}}_{\mathrm{BS}}$) $\overset{?}{=}$ 1	Global
OEMVerify (pbk, cert, ${\mathrm{pbk}}_{\mathrm{SD}}$)	Outputs 1 iff SigVerify ([pbk, ’Secure device’ || M], cert, ${\mathrm{pbk}}_{\mathrm{SD}}$) $\overset{?}{=}$ 1	Global
$T_{A}$.Deposit (x, …)	Deposits x amount of money into client A’s secure hardware $T_{A}$	$T_{A}$
$T_{A}$.Withdraw (x, …)	Withdraws x amount of money from client A’s secure hardware $T_{A}$	$T_{A}$
$T_{A}$.Pay (x, …)	Debits x amount of money from $T_{A}$ and outputs a payment P	$T_{A}$
$T_{A}$.Collect (P)	Credits a payment P into $T_{A}$’s balance	$T_{A}$

**Table 3:** Key protocols and functions executed by the ElasticPay trusted application (T) in the user’s TEE.

Init (): $(T.\mathrm{pbk},\;T.\mathrm{prk})\leftarrow \mathrm{KeyGen}(1^{\lambda })$ T.bal←0 T.offCounter←0 T.cert←⊥ T.inPaymentLog←⊥ σ←TOS.Attest(T.pbk) Output(T.pbk,σ) CertInit(cert): IfTACertVerify(cert,T.pbk)≠1 thenabort T.cert←cert	Deposit(x,BS.onCounter,σ): IfT.cert=⊥or BS.onCounter≠T.offCounter+1or $\mathrm{SigVerify}\;([x,\;\mathrm{BS}.\mathrm{onCounter}],\;\sigma ,{\mathrm{pbk}}_{\mathrm{BS}})$ ≠1thenabort T.offBal←T.offBal+x T.offCounter←T.offCounter+1
Withdraw(k): IfT.cert=⊥or k>T.offBalthenabort T.offBal←T.offBal−k T.offCounter←T.offCounter+1 Output[k,T.offCounter,σ] where σ←Sign([k,T.offCounter],T.prk)	Pay(x,timestamp,receiver): IfT.cert=⊥or T.offBal<xor SigVerify([timestamp,x],σ, receiver.pbk)≠1 thenabort T.offBal←T.offBal−x T.offCounter←T.offCounter+1 P.amount←x P.timestamp←timestamp P.index←T.offCounter P.sender←T.cert P.receiver←receiver P.sig←Sign(P,T.prk)OutputP
Collect(P): IfT.cert=⊥or PayVerify(P)≠1or P.receiver≠T.certor P∈T.inPaymentLog thenabort T.offBal←T.offBal+P.amount AppendPtoT.inPaymentLog	PrepareTxn(x): IfT.cert=⊥thenabort receiver←T.cert Output[[timestamp,x],σ,receiver] whereσ← $\mathrm{Sign}(\left[\mathrm{timestamp},\;x\right],T_{B}.\mathrm{prk})$ Get−Balance(): IfT.cert=⊥thenabort Output[T.offBal,T.offCounter,σ] whereσ← Sign([T.offBal,T.offCounter],T.prk)

**Table 4:** Transaction latency comparison—basic transaction system vs. ElasticPay.

Number of Transactions	Basic Transaction System Latency (s)	ElasticPay System Latency (s)
100,000	16.97	39.51
500,000	83.00	224.08
1,000,000	160.00	444.17

**Table 5:** Comparison of offline payment systems.

Ref.	Approach	Privacy Concerns	Issues Found Using the Approach
Christodorescu et al. [11]	TEE-based	Transaction count visible	Vulnerable during boot and foundation of trust undermined
Videira [13]	TEE-based	Full history required	Vulnerable during boot and foundation of trust undermined
Yang et al. [12]	TEE and SE	History shared	Vulnerable during boot and foundation of trust established
ElasticPay	TPM, TEE, and SE	Only necessary details required for the transaction	mitigates TEE boot vulnerability and foundation of trust established

**Table 6:** Mapping of ElasticPay features to security requirements.

Security Requirement	ElasticPay Mechanism	ElasticPay’s Solution
Prevention of Double-Spending	TEE-Based Token Generation and Validation	Transactions are processed inside a Trusted Execution Environment (TEE), ensuring that the transaction token can only be generated and used according to the correct procedure. If user ‘x’ attempts to reuse funds, they can only do so outside of the TEE by sending the same token generated by the TEE. However, the TEE of the original receiver ‘y’ will reject the transaction because it detects that the same token contains an identical Unix timestamp. If user ‘x’ tries to modify the timestamp while keeping the rest of the token unchanged, the signature will no longer match. Similarly, if user ‘x’ sends the altered token to another receiver ‘z’, the TEE of receiver ‘z’ will also reject the transaction due to the mismatch in the signature, making it impossible for the token to be altered or reused.
Unforgeability	TEE Isolation and Secure Signing	The private key used for signing transactions is securely stored exclusively within the TEE, making it inaccessible outside the trusted environment. Unless the private key is compromised, it is nearly impossible to forge a transaction, as the signing process can only occur within the TEE under genuine conditions.
Non-repudiation	Digital Signatures and Transaction Logging	ElasticPay ensures non-repudiation by using digital signatures generated within the TEE for each transaction. Since the signing process is controlled and isolated in the TEE, participants cannot deny their involvement in a transaction, providing cryptographic proof of each action.
Verifiability	Immutable Ledger and Token Transparency	The public ledger ensures that all transactions are verifiable. Each token, processed inside the TEE, contains essential details (recipient, amount, etc.), allowing third parties to independently verify transaction integrity without tampering.
Maintaining Anonymity	Pseudonymity via Public Keys & Cryptographic Privacy Mechanisms	ElasticPay ensures pseudonymity by using public keys to represent users instead of their real identities. Sensitive data are cryptographically protected, ensuring privacy during transactions. Users can change public keys for enhanced privacy while complying with KYC requirements for identity verification.

## Data Availability

Data are contained within the article.
